# The effect of job stress on smoking and alcohol consumption

**DOI:** 10.1186/2191-1991-1-15

**Published:** 2011-09-30

**Authors:** Sunday Azagba, Mesbah F Sharaf

**Affiliations:** 1 Department of Economics, Concordia University, 1455 de Maisonneuve Blvd. West Montréal, Quebec, H3G 1M8, Canada

**Keywords:** Job stress, job strain, smoking intensity, alcohol consumption, unobserved heterogeneity, latent class model

## Abstract

This paper examines the effect of job stress on two key health risk-behaviors: smoking and alcohol consumption, using data from the Canadian National Population Health Survey. Findings in the extant literature are inconclusive and are mainly based on standard models which can model differential responses to job stress only by observed characteristics. However, the effect of job stress on smoking and drinking may largely depend on unobserved characteristics such as: self control, stress-coping ability, personality traits and health preferences. Accordingly, we use a latent class model to capture heterogeneous responses to job stress. Our results suggest that the effects of job stress on smoking and alcohol consumption differ substantially for at least two "types" of individuals, light and heavy users. In particular, we find that job stress has a positive and statistically significant impact on smoking intensity, but only for light smokers, while it has a positive and significant impact on alcohol consumption mainly for heavy drinkers. These results provide suggestive evidence that the mixed findings in previous studies may partly be due to unobserved individual heterogeneity which is not captured by standard models.

## 1. Background

The work environment has witnessed dramatic changes in recent years as a result of globalization, competition, technological advances and economic uncertainty. Working conditions are now characterized by a high work load, an effort-reward imbalance, less job security, and the continual need to update skills [1]. Consequently, there is a growing concern that the workplace has adverse effects on the physical and psychological well-being of workers [1,2]. Substantial economic losses have been attributed to work-related stress. For example, work stress costs employers over $300 billion in the U.S [3] and £25.9 billion in the U.K annually [4], whereas in Canada, work time lost due to stress costs $12 billion per year [5]. It has been reported that work stress is responsible for 19% of absenteeism cost, 40% of turnover cost and 60% of workplace accidents [6]. In addition, a growing body of research has linked chronic stress to a wide range of adverse health outcomes such as mental disorder, cardiovascular disease, anxiety, depression, hostility, heart attack, headaches, back pain and colorectal cancer [7-10]. In particular, studies show that stress can induce several unhealthy behaviors such as smoking and excessive alcohol use [3,11].

The adverse health effects due to tobacco and excessive alcohol use are well documented in the literature. Smoking is the leading preventable cause of disease and premature death in the world [12]. It is a major risk factor for many diseases such as heart attacks, strokes, chronic obstructive pulmonary disease, cardiovascular disease and cancer [13,14]. Each year, about 6 million deaths are due to tobacco use and, by 2030, tobacco-related deaths are expected to reach 8 million yearly [12]. Chronic alcohol abuse also has serious effects on physical and mental health and can as well lead to an increased risk of accidents and crimes. Long-term excessive use of alcohol can exacerbate some medical conditions and is associated with a high risk of morbidity and mortality [15,16].

The association between job stress and smoking/alcohol use can be explained mainly on two grounds. First, individuals can self-medicate stress-induced physiological effects (such as elevated cortisol, suppressed serotonic, and catecholamine secretion) by smoking/drinking to achieve internal stability (homeostasis) [17,18]. Alcohol and cigarettes could also be used as anti-anxiety or anti-depressant agents to relieve the impact of job stress [19]. Second, job stress can reduce an individual's self-control, which makes it difficult for current smokers/drinkers to quit or reduce smoking/drinking intensity and may induce former smokers/drinkers to relapse and start to smoke/drink again [18,20]. Given that smoking and drinking are usually initiated before joining the labor market, several studies report that the impact of job stress on smoking and drinking intensity is more important than its impact on smoking and drinking status [21-24].

Several theoretical frameworks have been developed to model the effect of job stress on workers' physical and mental health. According to Karasek's job strain model, the interaction of psychological job demands and decision latitude (skill discretion and decision authority) determines how the psychosocial work environment can affect a worker's health [2]. Based on this model, high job strain results from a combination of high psychological demands and low decision latitude.

Empirical evidence on the relationship between job strain and smoking intensity is inconclusive [25]. In some studies, smoking intensity is positively associated with job demands [26-29] and with job strain [21,28-30], while negatively associated with job control [28,29,31]. For example, in a Finnish study of 46,190 public sector employees, Kouvonen et al. [29] find that workers with high job strain are more likely to be smokers than workers in jobs with low strain. They also find a positive and significant association between high job strain and smoking intensity among smokers. However, other studies find no significant association between smoking intensity and job demand [23,32,33], job control [23,33] or job strain [23,32-34]. For example, in a cross-sectional study of 6,995 white collar workers in 21 organizations, Brisson et al. [33] find no consistent association between smoking prevalence or intensity and high job strain. In a study of 3,701 Dutch workers, Otten et al. [32] find no significant association between job strain or high job demands and smoking behavior among men or women. However, they find a significant association for job control and smoking behavior, but only for men.

Findings from previous studies investigating the impact of job strain on alcohol consumption are similarly mixed [25]. While some studies find a positive association between job strain, or any of its components, and alcohol consumption [26,28,35,36], other studies find no relationship [23,34,37-39]. In a prospective cohort study, Van Loon et al. [40] examine the cross-sectional associations between job strain and several lifestyle risk factors for cancer, including smoking and alcohol consumption, low intake of fruit and vegetables, and physical inactivity. They find no statistically significant associations between any of the cancer-related lifestyles and job strain. However, in another study, San Jose et al. [36] find that stressful working conditions are positively associated with heavy and binge drinking in both men and women. Using a random sample of households in five metropolitan areas in the United States, Muntaner et al. [41] find a higher risk of drug abuse/dependence in individuals with high strain jobs and in individuals with high levels of physical demands and decision authority.

We propose that the mixed findings in the extant literature that examined the relationship between job stress and health-risk behaviors (smoking/drinking) may in part be due to unobserved characteristics that are not fully captured using standard models. Previous studies use models that estimate the average population response to job strain which may not necessarily be equal for all individuals. Even sample splitting by observed covariates in standard models (e.g., OLS, Poisson and logistic regression) cannot capture differential responses which are due to unobserved characteristics [42]. Moreover, most previous studies use a one-period (cross sectional) measure of job strain which may only reflect temporary effects, or small samples that are not necessarily representative of the population, while other studies focus only on some stressful occupations.

The objective of this paper is to examine the effect of job-related stress on the intensity of smoking and alcohol consumption. Job stress is measured by the Karasek's job strain model (high job demands and low job control) [2]. We use a latent class model (LCM) to capture population unobserved heterogeneity, and examine whether there are differences in behavioral responses to job strain. The latent class framework, unlike the standard models, is able to unmask hidden or complex relationships. Our findings indicate that the effects of job strain on smoking and alcohol consumption substantially differ for at least two "types" of individuals, light cigarette/alcohol users and heavy cigarette/alcohol users.

The rest of this paper is structured as follows: Section 2 describes the data; Section 3 presents the empirical method; results are discussed in Section 4; Section 5 presents further general discussion while the conclusions are summarized in Section 6.

## 2. Data

This study uses data from the Canadian National Population Health Survey (NPHS). The NPHS is a nationally representative sample of the Canadian population which collects vital information on health related behavior, as well as corresponding economic and socio-demographic variables. The survey excludes those living on Indian Reserves and Crown Lands, full-time members of the Canadian Forces and some remote areas of Ontario and Quebec.

The NPHS commenced in 1994/95 with a subsequent follow up every two years. Since the first cycle, there have been seven follow-up surveys, and cycle eight (2008/09) is currently available. The first cycle contains responses from 17,276 individuals. Since the main independent variable of interest, job strain, is not available in cycles two and three, this study uses data from cycle four (2000/01) to cycle eight (2008/09).

The outcome variables are daily smoking intensity (number of cigarettes) and alcohol consumption (number of drinks). We restrict the sample to those 18-65 years old since the smoking rate of those greater than 65 years is relatively small and also their health related issues may further complicate the analysis. Job strain, the main independent variable of interest, is an index that is derived from job related questions on decision latitude (skill discretion and decision authority) together with psychological demands. It is measured as a ratio of psychological demands and decision latitude, where higher values indicate greater job strain [2]. We stratify individuals based on the distribution of scores into tertiles to represent low, medium, and high levels of strain.

The study follows standard practice in the tobacco and alcohol literature by using a number of control variables. Real cigarette taxes, which include both the provincial and federal components, are included in the estimation. Age has three categories: 18-29 (reference category), 30-44, and 45-65. Household income is represented by four dummy variables: low income, low-middle income, high-middle income (reference category), and high income (see Table 1).

**Table 1:** Income categories based on NPHS classification

	Income	Household Size
Low income	Less than $15,000	1 or 2 persons
	Less than $20,000	3 or 4 persons
	Less than $30,000	5 or more persons
Low middle income	$15,000 to $29,999	1 or 2 persons
	$20,000 to $39,999	3 or 4 persons
	$30,000 to $59,999	5 or more persons
High middle income	$30,000 to $59,999	1 or 2 persons
	$40,000 to $79,999	3 or 4 persons
	$60,000 to $79,999	5 or more persons
High income	$60,000 or more	1 or 2 persons
	$80,000 or more	3 persons or more

Source: NPHS Household Component, Cycle 8 (2008/2009)

This classification is based on total household income and the number of people living in the household (for a detailed description, see [43]). Gender is captured by a dummy variable (male = 1, female = 0). Four dummy variables represent individual's educational attainment: less than secondary, secondary, some post secondary (reference category), and post secondary.

Marital status is represented by three dummy variables: married, separated and single (reference category). Household size is the family size. Ethnicity is captured by a dummy variable (immigrant = 1, Canadian born = 0). Workplace smoking restriction is represented by three categories: no ban (reference category), partial ban (smoking allowed in designated areas), and full ban. We include a measure of social support in the workplace since it has been suggested as an important stress modifier [44]. A higher social support score indicates lower workplace support.

Health status is represented by individual health utility index (HUI). The HUI is a set of generic, preference-based systems for measuring health status developed by the health utilities group at McMaster University. The index is constructed based on several dimensions of health status such as vision, hearing, speech, mobility, pain, dexterity, self-care, emotion and cognition. Each dimension has a score based on preference measurements from random samples of the general population [43,45]. Studies have validated the HUI as a more objective measure of individual health status than the commonly used self-rated health [46].

Provincial dummy variables are included with British Colombia as the reference category. To control for job-specific factors other than job strain which can affect smoking and alcohol consumption, seven occupational categories are extracted from the 2007 North American Industry Classification System available in the NPHS. We classify an individual's occupation into one of seven groups: mechanical, trade, professional, managerial, health, service, and farm (reference category). A linear time trend is included in all regression estimations. Table 2 provides a complete definition of the variables used in the analysis.

**Table 2:** Variables definition

Variable	Definition
Quantity(cigarette)	Daily number of cigarette smoked
Quantity(alcohol)	Daily number of drinks
Low strain	= 1 if job strain score belongs to the first quantile, 0 otherwise
Medium strain	= 1 if job strain score belongs to the second quantile, 0 otherwise
High strain	= 1 if job strain score belongs to the third quantile, 0 otherwise
Real cigarette tax	= Real excise cigarette tax per carton
Trend	= Linear year trend
Male	= 1 if gender is male, 0 otherwise
Female	= 1 if gender is female, 0 otherwise
Married	= 1 if married/living with a partner/common-law, 0 otherwise
Separated	= 1 if widowed/separated/divorced, 0 otherwise
Single	= 1 if never married, 0 otherwise (base category)
Less than secondary	= 1 if education is less than secondary, 0 otherwise
secondary	= 1 if education is secondary, 0 otherwise
Some post secondary	= 1 if education is some post secondary, 0 otherwise
Post secondary	= 1 if education is post secondary, 0 otherwise
Age 18-29	= 1 if aged 18-29 years, 0 otherwise
Age 30-44	= 1 if aged 30-44 years, 0 otherwise
Age 45-65	= 1 if aged 45-65 years, 0 otherwise
Low income	= 1 if household income is in low income group, 0 otherwise
Low middle income	= 1 if household income is in middle low income group, 0 otherwise
High middle income	= 1 if household income is in middle high income group, 0 otherwise
High income	= 1 if household income in high income group, 0 otherwise
Household size	= Number of people living in a household
Non immigrant	= 1 if country of birth is Canada, 0 otherwise
Immigrant	= 1 if country of birth is not Canada, 0 otherwise
No ban	= 1 if there is no workplace restrictions on smoking,0 otherwise
Partial ban	= 1 if smoking is allowed in designated areas,0 otherwise
Full ban	= 1 if there is full workplace restrictions on smoking,0 otherwise
Social support	Social support score, indicating the social support available to the respondent at his/her main job in the past 12 months.
HUI	Health utility index
Newfoundland	= 1 if province of residence is Newfoundland, 0 otherwise
Prince Edward	= 1 if province of residence is Prince Edward, 0 otherwise
Nova Scotia	= 1 if province of residence is Nova Scotia, 0 otherwise
New Brunswick	= 1 if province of residence is New Brunswick, 0 otherwise
Quebec	= 1 if province of residence is Quebec, 0 otherwise
Ontario	= 1 if province of residence is Ontario, 0 otherwise
Manitoba	= 1 if province of residence is Manitoba, 0 otherwise
Saskatchewan	= 1 if province of residence is Saskatchewan, 0 otherwise
Alberta	= 1 if province of residence is Alberta, 0 otherwise
British Columbia	= 1 if province of residence is British Columbia, 0 otherwise
Mechanical	= 1 if individual's job belong to mechanical occupations,0 otherwise
Trade	= 1 if individual's job belong to trade occupations,0 otherwise
professional	= 1 if individual's job belong to professional occupations,0 otherwise
managerial	= 1 if individual's job belong to managerial occupations,0 otherwise
Health	= 1 if individual's job belong to health occupations,0 otherwise
Service	= 1 if individual's job belong to services occupations,0 otherwise
Farm	= 1 if individual's job belong to farm occupations,0 otherwise

## 3. Methods

To examine the relationship between job strain, smoking and alcohol consumption, the following reduced-form model is estimated:

(1) $$y_{ijt}=\gamma {\left(jobstrain\right)}_{it}+\beta^{\prime }X_{it}+\delta J_{t}+\theta Q_{jt}+\varphi {\left(OC\right)}_{it}+\varepsilon_{ijt}$$

where *i *indicates the individual, *j *represents province of residence, and *t *represents the year, *y *represents the daily number of cigarettes and alcohol drinks consumed. *jobstrain *represents the three categories of strain levels, ***X ***is a vector of other control variables including: cigarette taxes, age, income, gender, household size, employment status, education, marital status, workplace social support, workplace smoking restrictions, and ethnicity. *J *represents a linear time trend. The province fixed-effect variable, *Q*, is included to capture smoking ban regulations and other cultural factors that may be region-specific. In Canada, the Municipal Act 2001 enables municipalities to enact by-laws like smoking bans or restrictions in public places. OC represents occupational classifications and ε*_ijt _*is the standard time variant residual term which is adjusted for clustering at the individual level.

We begin our analysis by using conventional econometric models (OLS, Poisson, and the negative binomial) to estimate Equation (1). These standard specifications produce a one population estimate of the job strain coefficient, γ, by assuming that the impact of job strain on smoking/alcohol consumption is equal for all individuals. While in some instances this generalization may be correct, it will be misleading if the population is characterized by distinct subpopulations. In particular, responses to job strain could likely depend on unobserved characteristics such as: self control, stress coping ability, health preference, personality (e.g. neuroticism) and other "decision-making characteristics" [[47], pg. 8]. It has been argued that personality traits can play a significant role in the way people perceive and react to stress [1]. Accordingly, we estimate Equation (1) using a latent class framework to account for individual unobserved heterogeneity in response to job strain.

The latent class model splits the population into subpopulations of different types -in this case-, light or heavy smokers and drinkers according to an individual's latent status. In this model, the dependent variable, *y*, comes from a population that comprises *C *distinct subpopulations, with unknown missing weights *π*_1_,... *π_C _*where 0 ≤ *π_j _*≤ 1 and $\sum_{j=1}^{C}\pi_{j}=1$. The finite mixture density of *y *with *C *support points is given by

(2) $$f\left(y_{i}|\Theta \right)=\overset{C-1}{\underset{j=1}{\sum }}\pi_{j}f_{j}\left(y_{i}|\theta_{j}\right)+\pi_{C}f_{C}\left(y_{C}|\theta_{C}\right)$$

where the mixing weights (probabilities), *π_j_*, are estimated along with the other parameters, denoted **Θ**. The *C *point latent negative binomial distributions are specified as

(3) $$f_{j}\left(y_{i}\right)=\frac{\Gamma \left(y_{i}+\psi_{j,i}\right)}{\Gamma \left(\psi_{j,i}\right)\Gamma \left(y_{i}+1\right)}{\left(\frac{\psi_{j,i}}{\lambda_{j,i}+\psi_{j,i}}\right)}^{\psi_{j,i}}{\left(\frac{\lambda_{j,i}}{\lambda_{j,i}+\psi_{j,i}}\right)}^{y_{i}}$$

where $\lambda_{\text{j,i}}=\exp\left(\chi_{\text{i}}^{\prime }\beta_{\text{j}}\right),\;\Gamma \left(\cdot \right)$ is the gamma function and $\psi_{\text{j,i}}=\left(1∕\alpha_{\text{j}}\right)\lambda_{\text{j,i}}^{\text{k}}.$ In this study, we use the Poisson (*i.e*. the dispersion paremeter, α = 0) and negative binomial 2 (*i.e*. α > 0 &*k *= 0) variant for the mixture component densities. Other advantages of using the latent class framework have been documented in the literature: (a) it enables unobserved heterogeneity to be captured in a simple and intuitive way; (b) it is semi-parametric, since the mixing variable is not distribution specific; (c) it is valid even if the underlying mixing distribution is continuous (d) usually two or three points are sufficient to approximate the mixing distribution; and (e) some continuous mixing models may not have a closed-form solution [48,49].

In health-related outcomes, the use of a latent class framework is even more appealing given that an individual's observed characteristics may not reflect their long-term health preferences [49,50]. Following previous studies, we hypothesize that individuals' unobserved health attitudes are captured by a finite mixture distribution which splits the population into two distinct classes of smokers and drinkers [48-50]. We estimate a two latent components negative binomial model for smoking and a two latent components Poisson model for alcohol consumption. We classify the two components into a light-use group, on the basis of low predicted mean, and a heavy-use group, with a high predicted mean.

## 4. Results

The Summary statistics for the variables used in the analysis are reported in Table 3. On average, smokers consume 12.8 cigarettes per day and drinkers consume 0.6 drinks per day. About one third of the sample work in jobs with high strain while a quarter works in jobs with medium strain. On average, the health utility index of Canadian adult workers of more than 0.9 indicates a good functional health. Household size is 3 on average. 49% of the Canadian workers have full bans on smoking in the workplace whereas 37% have partial bans. 55% of the smoking sample is male, 54% are married, 68% have postsecondary education or above and 10% are immigrants. For the alcohol consumption sample, 53% is male, 63% is married, 77% have at least a postsecondary education and 14% are immigrants.

**Table 3:** Descriptive statistics.

	**Smoking**		**Alcohol**	
	
**Variables**	**Mean**	**S.D**	**Mean**	**S.D**
Quantity	12.845	0.099	0.617	0.007
High strain	0.372	0.005	0.314	0.003
Medium strain	0.231	0.005	0.235	0.003
Low strain	0.397	0.006	0.412	0.003
Male	0.554	0.006	0.533	0.003
Female	0.446	0.006	0.467	0.003
Married	0.540	0.006	0.628	0.003
Separated	0.144	0.004	0.102	0.002
Single	0.316	0.005	0.269	0.003
Less secondary	0.152	0.004	0.096	0.002
Secondary	0.171	0.004	0.135	0.002
Some postsecondary	0.313	0.005	0.283	0.003
postsecondary	0.364	0.005	0.485	0.003
Age 18-29	0.294	0.005	0.249	0.003
Age 30-44	0.363	0.005	0.359	0.003
Age 45-65	0.343	0.005	0.392	0.003
Low income	0.047	0.002	0.030	0.001
Low middle income	0.150	0.004	0.108	0.002
High middle income	0.354	0.005	0.320	0.003
High income	0.375	0.005	0.475	0.003
Household size	2.900	0.015	3.067	0.008
Non immigrant	0.897	0.003	0.857	0.002
immigrant	0.103	0.003	0.142	0.002
No ban	0.138	0.004	-	-
Partial ban	0.367	0.005	-	-
Full ban	0.492	0.006	-	-
Social support	4.192	0.022	4.014	0.012
HUI	0.907	0.002	0.923	0.001
Newfoundland	0.015	0.001	0.016	0.001
Prince Edward	0.006	0.001	0.005	0.000
Nova Scotia	0.033	0.002	0.030	0.001
New Brunswick	0.023	0.002	0.022	0.001
Quebec	0.265	0.005	0.257	0.003
Ontario	0.369	0.005	0.372	0.003
Manitoba	0.035	0.002	0.035	0.001
Saskatchewan	0.034	0.002	0.032	0.001
Alberta	0.119	0.004	0.109	0.002
British Colombia	0.103	0.003	0.116	0.002
Mechanical	0.221	0.005	0.191	0.002
Trade	0.216	0.005	0.193	0.002
Professional	0.123	0.004	0.143	0.002
Managerial	0.143	0.004	0.172	0.002
Health	0.085	0.003	0.113	0.002
Service	0.167	0.004	0.144	0.002
Farm	0.040	0.002	0.040	0.001
N	7880		27063	

The statistics are weighted using the NPHS sampling weights.

First, we present results from the traditional model with an average population estimates for the effect of strain on cigarettes consumption in Table 4. Only the OLS results are reported here since we find that there are no significant differences between it and the Poisson and negative binomial models (all results are available upon request from the authors). Next, the LCM results enable us to examine whether there exists a differential health behavior response to job strain. Our results support the presence of at least two distinct latent classes of smokers/drinkers. These results emphasize the importance of controlling for unobserved heterogeneity in estimating the effect of job strain on smoking and alcohol consumption.

**Table 4:** OLS model for smoking: daily number of cigarette consumption

	Model 1	Model 2	Model 3
High strain	1.328***	1.154***	1.026***
	(0.278)	(0.276)	(0.274)
Medium strain	0.567**	0.457*	0.379
	(0.254)	(0.254)	(0.254)
Real cigarette tax	-0.047***	-0.046***	-0.028*
	(0.017)	(0.017)	(0.017)
Trend	-0.121**	-0.128***	-0.191***
	(0.048)	(0.049)	(0.047)
Male	2.821***	2.781***	2.717***
	(0.300)	(0.296)	(0.306)
Married	0.339	0.288	0.309
	(0.337)	(0.337)	(0.335)
Separated	2.010***	1.919***	1.973***
	(0.493)	(0.484)	(0.487)
Less secondary	2.019***	1.951***	1.773***
	(0.440)	(0.435)	(0.445)
Secondary	0.352	0.310	0.231
	(0.433)	(0.432)	(0.430)
Post secondary	-0.608*	-0.642*	-0.621*
	(0.356)	(0.350)	(0.349)
Age 30-44	3.190***	3.202***	3.183***
	(0.338)	(0.338)	(0.340)
Age 45-65	5.064***	5.069***	4.957***
	(0.404)	(0.401)	(0.404)
Low income	0.531	0.460	0.205
	(0.475)	(0.470)	(0.476)
Low middle income	0.758**	0.837***	0.624**
	(0.307)	(0.305)	(0.312)
High income	-0.900***	-0.828***	-0.616**
	(0.290)	(0.284)	(0.285)
Household size	-0.126	-0.123	-0.105
	(0.112)	(0.111)	(0.111)
Immigrant	-2.878***	-2.887***	-2.638***
	(0.594)	(0.593)	(0.597)
Partial ban	-1.864***	-1.901***	-1.817***
	(0.392)	(0.392)	(0.397)
Full ban	-3.349***	-3.441***	-3.347***
	(0.399)	(0.403)	(0.415)
Social support		0.157***	0.094
		(0.058)	(0.058)
HUI			-4.649***
			(0.922)
Newfoundland			0.353
			(0.914)

Prince Edward			1.642**
			(0.731)
Nova Scotia			0.671
			(0.765)
New Brunswick			1.788**
			(0.813)
Quebec			1.552**
			(0.625)
Ontario			0.474
			(0.612)
Manitoba			0.680
			(0.780)
Saskatchewan			1.152
			(0.725)
Alberta			0.696
			(0.619)
Mechanical			-0.047
			(0.647)
Trade			0.103
			(0.664)
Professional			-0.877
			(0.714)
managerial			-0.456
			(0.694)
Health			-0.452
			(0.750)
Service			-0.018
			(0.666)
Constant	12.470***	12.010***	15.410***
	(0.735)	(0.782)	(1.422)
			
Observations	7880	7763	7696

Robust standard errors in parentheses. *** p < 0.01, ** p < 0.05, * p < 0.1. Model 1 presents the baseline specification. An additional covariate, workplace social support, is added in model 2. In model 3, we add individual's health status (HUI), province of residence and occupational fixed effects.

### 4.1 Smoking results

The single equation OLS (no latent subgroups) model for cigarette consumption with different specifications is reported in Table 4. Model 1 presents the baseline specification. An additional covariate, workplace social support, is added in model 2. In model 3, we add individual's health status (HUI), province of residence and occupational fixed effects. We also excluded occupational categories in a different specification (unreported, but available on request), but there was no effect on the results. We find that high job strain has a positive and significant effect on smoking intensity compared to low job strain and this result is robust to models 2 & 3 specifications. The inclusion of workplace social support, which acts as a stress modifier, is significant in model 2 and thus reduces the impact of job strain. Note that the positive sign of the social support coefficient indicates that a low social support is associated with high smoking intensity. This is due to the way social support index is defined, where a high value indicates low workplace social support. The impact of medium job strain is similar except for model 3, where it has no significant effect on smoking intensity. Other variables included in the model have the expected signs. The socioeconomic variables (SES) confirm the standard SES smoking gradient: those with more education and income tend to smoke less. The real cigarette tax has a moderate negative impact, and males smoke more than females. Immigrants smoke less than natives and workplace smoking restrictions have a negative and significant effect on the quantity smoked.

In Table 5, we present results from the LCM which examines differential responses to job strain based on unobserved individual characteristics. The results indicate a substantial difference between the two latent classes. In particular, we find that a large group (over 70%) is light smokers and the effect of high job strain is positive and significant for this group. The estimates for the effect of high job strain for the group of heavy smokers are considerably smaller and not statistically significant. These results are also robust to the inclusion of other variables in models 2 & 3. Similar findings of positive and significant effects are obtained for medium job strain except for model 3. The impact of the other control variables is generally similar to the OLS results.

**Table 5:** Latent class model for smoking: daily number of cigarette consumption

	Model 1		Model 2		Model 3	
	
	Comp1	comp2	comp1	Comp2	Comp1	Comp2
High strain	0.116***	0.016	0.111***	0.002	0.102***	-0.009
	(0.027)	(0.033)	(0.027)	(0.033)	(0.028)	(0.031)
Medium strain	0.061**	-0.007	0.056**	-0.013	0.045	-0.013
	(0.027)	(0.026)	(0.027)	(0.026)	(0.027)	(0.026)
Real cigarette tax	-0.002	-0.004*	-0.003	-0.004*	-0.002	-0.0003
	(0.002)	(0.002)	(0.002)	(0.002)	(0.002)	(0.002)
Trend	-0.013**	-0.0001	-0.013**	-0.001	-0.017***	-0.009
	(0.005)	(0.006)	(0.005)	(0.006)	(0.005)	(0.006)
Male	0.144***	0.221***	0.141***	0.221***	0.132***	0.214***
	(0.036)	(0.073)	(0.034)	(0.060)	(0.033)	(0.063)
Married	0.029	0.008	0.025	0.002	0.001	0.042
	(0.038)	(0.043)	(0.038)	(0.041)	(0.037)	(0.051)
Separated	0.169***	0.066	0.164***	0.064	0.150***	0.099**
	(0.049)	(0.048)	(0.049)	(0.050)	(0.047)	(0.049)
Less seconday	0.177***	0.059	0.177***	0.048	0.160***	0.030
	(0.043)	(0.044)	(0.043)	(0.044)	(0.044)	(0.050)
Secondary	0.028	0.056	0.024	0.052	0.009	0.052
	(0.042)	(0.052)	(0.042)	(0.051)	(0.042)	(0.057)
Post secondary	-0.102***	0.043	-0.104***	0.036	-0.096***	0.037
	(0.037)	(0.042)	(0.037)	(0.041)	(0.036)	(0.045)
Age 30-44	0.261***	0.234***	0.265***	0.230***	0.271***	0.212***
	(0.039)	(0.058)	(0.039)	(0.048)	(0.039)	(0.055)
Age 45-65	0.385***	0.322***	0.389***	0.321***	0.392***	0.281***
	(0.045)	(0.051)	(0.045)	(0.049)	(0.045)	(0.057)
Low income	0.048	0.063	0.051	0.043	0.015	0.048
	(0.048)	(0.075)	(0.048)	(0.076)	(0.049)	(0.079)
Low middle income	0.072**	0.020	0.081***	0.020	0.057*	0.017
	(0.031)	(0.028)	(0.031)	(0.028)	(0.030)	(0.029)
High income	-0.085***	-0.023	-0.078**	-0.031	-0.057*	-0.018
	(0.031)	(0.030)	(0.031)	(0.029)	(0.030)	(0.036)
Household size	-0.011	-0.003	-0.011	-0.003	-0.008	-0.006
	(0.012)	(0.011)	(0.012)	(0.011)	(0.012)	(0.011)
Immigrant	-0.296***	-0.008	-0.300***	-0.024	-0.273***	-0.057
	(0.057)	(0.109)	(0.058)	(0.102)	(0.061)	(0.089)
Partial ban	-0.103***	-0.100***	-0.106***	-0.100***	-0.102***	-0.081**
	(0.035)	(0.031)	(0.036)	(0.031)	(0.036)	(0.036)
Full ban	-0.250***	-0.137***	-0.255***	-0.148***	-0.244***	-0.141***
	(0.037)	(0.045)	(0.038)	(0.039)	(0.038)	(0.048)
Social support			0.007	0.015**	0.004	0.011
			(0.006)	(0.006)	(0.006)	(0.006)
HUI					-0.274***	-0.224*
					(0.091)	(0.124)
Newfoundland					0.044	0.006
					(0.089)	(0.162)

Prince Edward					0.119	0.128
					(0.075)	(0.118)
Nova Scotia					0.011	0.147
					(0.077)	(0.102)
New Brunswick					0.209***	-0.002
					(0.079)	(0.130)
Quebec					0.076	0.215**
					(0.067)	(0.105)
Ontario					0.010	0.127
					(0.065)	(0.110)
Manitoba					-0.018	0.174
					(0.080)	(0.130)
Saskatchewan					0.075	0.070
					(0.076)	(0.113)
Alberta					0.063	0.059
					(0.066)	(0.112)
Mechanical					0.019	-0.059
					(0.069)	(0.050)
Trade					0.014	-0.018
					(0.060)	(0.051)
Professional					-0.090	-0.034
					(0.071)	(0.071)
Managerial					-0.081	0.050
					(0.066)	(0.057)
Health					-0.076	0.026
					(0.072)	(0.097)
Service					-0.001	-0.016
					(0.064)	(0.056)
Constant	2.340***	2.766***	2.315***	2.711***	2.533***	2.770***
	(0.078)	(0.101)	(0.086)	(0.102)	(0.152)	(0.211)
π_1_	0.729 (0.056)	0.271	0.722 (0.044)	0.278	0.746 (0.056)	0.254
Observations	7880	7880	7763	7763	7696	7696

Robust standard errors in parentheses; π_1 _stands for the probability that an observation is in comp1; *** p < 0.01, ** p < 0.05, * p < 0.1. Model 1 presents the baseline specification. An additional covariate, workplace social support, is added in model 2. In model 3, we add individual's health status (HUI), province of residence and occupational fixed effects.

### 4.2 Alcohol consumption results

As with cigarette consumption, single equation (no latent subgroups) OLS estimates of the job strain effects on the intensity of drinking are reported in Table 6. In all model specifications, the coefficient of high job strain is not statistically significant. Also, medium job strain has no significant effect on alcohol consumption except for model 1. The effects of other variables in the model are somewhat similar to the cigarette results presented above. Being immigrant, married, more educated and older significantly reduces the number of drinks consumed. The impact of household size is also negative and significant. Those in the high income category drink more. Some of the provincial and occupation variables are also significant.

**Table 6:** OLS model for daily alcohol consumption

	Model 1	Model 2	Model 3
High strain	0.007	-0.007	-0.010
	(0.014)	(0.014)	(0.014)
Medium strain	0.031**	0.020	0.019
	(0.015)	(0.016)	(0.016)
Trend	0.010***	0.009***	0.009***
	(0.002)	(0.002)	(0.002)
Male	0.485***	0.480***	0.462***
	(0.012)	(0.012)	(0.013)
Married	-0.142***	-0.137***	-0.122***
	(0.018)	(0.018)	(0.018)
Separated	0.012	0.017	0.018
	(0.024)	(0.025)	(0.025)
Less secondary	-0.034	-0.027	-0.038
	(0.024)	(0.024)	(0.025)
secondary	0.041*	0.051**	0.041*
	(0.022)	(0.022)	(0.022)
Post secondary	-0.040***	-0.044***	-0.034**
	(0.014)	(0.014)	(0.014)
Age 30-44	-0.113***	-0.120***	-0.116***
	(0.018)	(0.019)	(0.019)
Age 45-65	-0.099***	-0.112***	-0.106***
	(0.020)	(0.020)	(0.020)
Low income	0.013	0.008	0.001
	(0.033)	(0.034)	(0.034)
Low middle income	-0.007	-0.018	-0.023
	(0.020)	(0.020)	(0.020)
High income	0.178***	0.178***	0.172***
	(0.013)	(0.014)	(0.014)
Household size	-0.021***	-0.020***	-0.022***
	(0.005)	(0.005)	(0.005)
immigrant	-0.147***	-0.140***	-0.173***
	(0.017)	(0.017)	(0.018)
Social support		0.009**	0.008**
		(0.004)	(0.004)
HUI			-0.075
			(0.052)
Newfoundland			-0.024
			(0.032)
Prince Edward			-0.099***
			(0.033)
Nova Scotia			-0.116***
			(0.029)
New Brunswick			-0.082***
			(0.030)

Quebec			-0.053**
			(0.023)
Ontario			0.012
			(0.023)
Manitoba			-0.053*
			(0.030)
Saskatchewan			-0.060**
			(0.031)
Alberta			-0.093***
			(0.025)
Mechanical			0.088***
			(0.033)
Trade			0.013
			(0.032)
Professional			0.022
			(0.032)
Managerial			0.020
			(0.031)
Health			-0.068**
			(0.031)
Service			0.143***
			(0.034)
Constant	0.466***	0.450***	0.538***
	(0.025)	(0.029)	(0.069)
			
Observations	27063	25637	25472

Robust standard errors in parentheses. *** p < 0.01, ** p < 0.05, * p < 0.1. Model 1 presents the baseline specification. An additional covariate, workplace social support, is added in model 2. In model 3, we add individual's health status (HUI), province of residence and occupational fixed effects.

The LCM results reported in Table 7 indicate significant heterogeneity between the two latent classes. The average daily drinking of one group is about five times as large as the other group. In particular, a small group (less than 11%) is heavy drinkers with an average of about 2.1 drinks per day while the large group (over 89%) is light drinkers with about 0.4 drinks. In contrast to the single equation results, we find a modest and statistically significant effect of job strain on drinking. The effect of high/medium strain is positive and significant for the heavy use group. It is only significant at a 10% significance level when workplace social support, health status, province and occupation variables are included in the model (see model 3). The coefficient of high job strain is negative for light drinkers and is also significant in models 2 & 3. This result may not be surprising since the average alcohol consumption for this group is relatively low; it is possible that light drinkers may self-medicate job stress by ways other than drinking (e.g., smoking and food). The effects of the other control variables are qualitatively similar to the OLS estimates.

**Table 7:** Latent class model for daily alcohol consumption

	Model 1		Model 2		Model 3	
	
	Comp1	comp2	comp1	Comp2	comp1	comp2
High strain	-0.041	0.131***	-0.067**	0.112**	-0.063**	0.092*
	(0.028)	(0.050)	(0.029)	(0.056)	(0.030)	(0.049)
Medium strain	0.028	0.108**	0.005	0.010*	0.002	0.099*
	(0.028)	(0.051)	(0.029)	(0.054)	(0.030)	(0.055)
Trend	0.014***	0.020**	0.012***	0.018**	0.013***	0.018**
	(0.004)	(0.008)	(0.004)	(0.008)	(0.004)	(0.008)
Male	0.852***	0.966***	0.835***	0.949***	0.785***	0.899***
	(0.027)	(0.054)	(0.028)	(0.056)	(0.031)	(0.061)
Married	-0.198***	-0.205***	-0.188***	-0.197***	-0.157***	-0.186***
	(0.032)	(0.063)	(0.032)	(0.065)	(0.034)	(0.061)
Separated	0.003	0.097	0.004	0.102	0.022	0.057
	(0.045)	(0.072)	(0.046)	(0.076)	(0.047)	(0.076)
Less secondary	-0.161***	0.008	-0.136***	0.004	-0.150***	-0.035
	(0.047)	(0.064)	(0.048)	(0.066)	(0.050)	(0.066)
secondary	-0.0001	0.123*	0.014	0.147**	0.012	0.097
	(0.040)	(0.065)	(0.040)	(0.066)	(0.042)	(0.061)
Post secondary	0.026	-0.270***	0.016	-0.273***	0.036	-0.241***
	(0.028)	(0.055)	(0.029)	(0.056)	(0.030)	(0.055)
Age 30-44	-0.178***	-0.217***	-0.192***	-0.213***	-0.183***	-0.196***
	(0.034)	(0.055)	(0.034)	(0.056)	(0.035)	(0.056)
Age 45-65	-0.010***	-0.236***	-0.121***	-0.251***	-0.105***	-0.209***
	(0.036)	(0.067)	(0.037)	(0.070)	(0.038)	(0.069)
Low income	0.023	0.056	0.022	0.058	0.017	0.101
	(0.075)	(0.105)	(0.076)	(0.112)	(0.078)	(0.117)
Low middle income	-0.122***	0.153***	-0.130***	0.116**	-0.141***	0.110**
	(0.046)	(0.057)	(0.048)	(0.058)	(0.049)	(0.056)
High income	0.408***	0.150***	0.410***	0.144***	0.406***	0.131**
	(0.028)	(0.053)	(0.029)	(0.055)	(0.031)	(0.053)
Household size	-0.036***	-0.053***	-0.034***	-0.053**	-0.036***	-0.067***
	(0.011)	(0.020)	(0.011)	(0.021)	(0.011)	(0.017)
immigrant	-0.240***	-0.336***	-0.226***	-0.307***	-0.258***	-0.365***
	(0.044)	(0.092)	(0.045)	(0.097)	(0.051)	(0.111)
Social support			-6.09e-05	0.030**	-0.002	0.027**
			(0.007)	(0.012)	(0.007)	(0.013)
HUI					0.177*	-0.340**
					(0.107)	(0.146)
Newfoundland					-0.043	-0.037
					(0.064)	(0.135)
Prince Edward					-0.307***	0.028
					(0.075)	(0.158)
Nova Scotia					-0.309***	-0.062
					(0.069)	(0.116)
New Brunswick					-0.203***	-0.126
					(0.067)	(0.118)

Quebec					-0.074	-0.061
					(0.046)	(0.109)
Ontario					-0.101**	0.192*
					(0.044)	(0.102)
Manitoba					-0.186***	0.090
					(0.064)	(0.120)
Saskatchewan					-0.192***	0.103
					(0.061)	(0.141)
Alberta					-0.302***	0.071
					(0.052)	(0.109)
Mechanical					0.094	0.132
					(0.059)	(0.084)
Trade					0.012	-0.004
					(0.060)	(0.086)
Professional					0.106*	-0.165
					(0.064)	(0.100)
Managerial					0.062	-0.074
					(0.061)	(0.093)
Health					-0.234***	-0.321**
					(0.074)	(0.132)
Service					0.237***	0.197**
					(0.063)	(0.095)
Constant	-1.282***	0.444***	-1.241***	0.338**	-1.326***	0.590**
	(0.055)	(0.115)	(0.061)	(0.133)	(0.138)	(0.236)
π_1_	0.905 (0.010)	0.095	0.903 (0.011)	0.097	0.890 (0.013)	0.11
Observations	27063	27063	25637	25637	25472	25472

Robust standard errors in parentheses; π_1 _stands for the probability that an observation is in comp1; *** p < 0.01, ** p < 0.05, * p < 0.1. Model 1 presents the baseline specification. An additional covariate, workplace social support, is added in model 2. In model 3, we add individual's health status (HUI), province of residence and occupational fixed effects.

## 5. Discussion

The individuals' differential responses to job stress can be explained on several grounds. Individuals have different preferences and hence may differ in the type of self medicating strategies they use to cope with stress. For example, some individuals may respond to stress by smoking more, while others may consume more food or alcohol [51]. This implies that the way individuals perceive and react to stress may vary with unobserved characteristics. These health risk behaviors could be substitutes for some individuals while for others they may be complementary stress relievers.

Some individuals, especially those whose consumption quantities are apparently not affected by stress, may engage in compensatory behaviors which are not reflected by the observed consumption quantities. For instance, smokers may consume cigarettes more intensively through increasing the number of puffs, length of inhalation, or by blocking the ventilation holes on the filter while consuming the same number of cigarettes [52]. We believe that this compensatory behavior is of particular importance when assessing the impact of stress on health risk behaviors. However, this behavior is not captured by the current study since there is no relevant information about it in the data set. Also, individuals may differ in their stress-tolerance level. In this case, any consumption pattern for alcohol/cigarette is possible. Parental and family background may influence the way offspring respond to stress [53].

The effects of job strain on smoking and alcohol consumption are quite different between the standard models and the latent class framework. This has some important policy implications. For example, in contrast to the OLS results, the latent class model indicates that job strain has a significant effect on alcohol consumption. Accordingly, policy intervention measures would not be necessary if based on the empirical results from the single equation model. The OLS results assume that the impact of job strain on smoking and alcohol consumption is equal on average for all users. This generalization would be wrong if the population is actually characterized by distinct subpopulations.

A "carpet bombing" intervention strategy is costly and likely less effective, if there are considerable differences in behavioral response to job strain. Therefore, identifying the group of smokers/drinkers who are more susceptible would help in designing more effective intervention measures. There is no "one-size fits all" strategy for discouraging health risk behaviors [54].

Our results show that job strain increases drinking intensity for heavy drinkers, while it increases smoking intensity for light smokers. Excessive use of alcohol has dangerous effects on physical and mental health, and increases the risk of morbidity and mortality [16]. Early intervention may prevent light smokers from getting addicted to smoking. In general, stress management and moves to relieve stressful working conditions should be an integral part of any smoking/drinking cessation program.

## 6. Conclusions

In this study, we use nationally representative data from the Canadian National Population Health Survey to assess the effect of job strain on two key health-risk behaviors: smoking and alcohol consumption. This study is motivated by the inconclusive findings in the related literature which are mainly based on the standard average population estimate models. The contribution of the current study to the literature is threefold. First, we use a measure of job strain that better represents individuals' long-term work conditions rather than the one-period (cross sectional) measure. Second, the use of latent class model which splits the population into subgroups enables us to study the heterogeneous responses of each group. Third, we compare the results from standard models to the latent class model. Our results provide suggestive evidence that the single equation models do not fully capture the relationship between job strain and health-risk behaviors and hence may partly account for the mixed findings in previous studies.

The results of this study indicate that among smokers, light users are the most vulnerable group. While for alcohol consumption, the effect of job strain is positive and significant mainly for heavy drinkers. One possible reason for the differential effects of job strain between light and heavy smokers may be due to the varying degree of sensitization to tobacco use among these groups. Since heavy smokers are already at higher levels of consumption, they may self-medicate stress through other ways (e.g. alcohol and food consumption). Our findings are robust to the inclusion of workplace social support, health status, province and occupation fixed effects. Results also reveal the importance of the workplace social support which acts as a stress modifier. The inclusion of the social support index reduced the impact of job strain. Workplace intervention measures may be beneficial, particularly for the high risk groups. Some intervention strategies have been shown to be effective [3,8,55,56]. For example, nicotine replacement therapy which promotes gradual withdrawal from the harmful effects of nicotine, health promotion/wellness programs, stress management programs (e.g. individual and group counseling), social support and employee assistance programs have all proven to be beneficial.

## Competing interests

The authors declare that they have no competing interests.

## Authors' contributions

Both authors helped in the conceptualization, design and write up of the manuscript. SA conducted the data analysis. Both authors read and approved the final draft.
